# Primary drug resistance of mycobacterium tuberculosis in Shandong, China, 2004–2018

**DOI:** 10.1186/s12931-019-1199-3

**Published:** 2019-10-18

**Authors:** Wan-mei Song, Yi-fan Li, Xiao-bin Ma, Jin-yue Liu, Ning-ning Tao, Yao Liu, Qian-yun Zhang, Ting-ting Xu, Shi-jin Li, Chun-Bao Yu, Lei Gao, Liang-liang Cui, Huai-chen Li

**Affiliations:** 1 Department of Respiratory Medicine, Shandong Provincial Hospital Affiliated to Shandong University, Jinan, 250021 Shandong China; 2 Cheeloo College of Medicine, Shandong University, Jinan, 250012 Shandong China; 3 School of Medicine and Life Sciences, University of Jinan-Shandong Academy of Medical Sciences, Jinan, 250200 Shandong China; 4 Peking Union Medical College, Beijing, 100005 China; 5 Department of Respiratory Medicine, Shandong Provincial Chest Hospital, Jinan, 250013 Shandong China; 6 NHC Key Laboratory of Systems Biology of Pathogens, Institute of Pathogen Biology, and Center for Tuberculosis Research, Chinese Academy of Medical Sciences and Peking Union Medical College, Beijing, 100730 China; 7 Department of Biostatistics, School of Public Health, Shandong University, Jinan, 250012 Shandong China; 8 Jinan Municipal Center for Disease Control and Prevention, Jinan, 250021 Shandong China; 9 Shandong University of Traditional Chinese Medicine, Jinan, 250355 Shandong China

**Keywords:** Tuberculosis, Primary drug resistance, Epidemiology, Risk factors, Joinpoint regression model

## Abstract

**Background:**

Primary drug-resistant tuberculosis (DR-TB) has contributed to a significant health and economic burden on a global scale, especially in China. we sought to estimate epidemiological characteristics of primary DR-TB in China from 2004 to 2018.

**Methods:**

Eleven thousand four hundred sixty-seven newly diagnosed and 1981 retreated TB cases with drug susceptibility data were included. Chi-Square test for trends, linear regression, a joinpoint regression model and temporal trend in proportions of the different resistance patterns were carried out.

**Results:**

The proportion of primary DR-TB and mono-resistant TB (MR-TB) in China had reduced by more than 12% since 2004, and were 21.38%, 13.35% in 2018 respectively. Among primary DR-TB cases (2173,18.95%), the percentage of multiresistant TB (MDR-TB, from 5.41 to 17.46%), male (from 77.03 to 84.13%), cavity (from 13.51 to 43.92%), rifampicin(RFP)-resistant TB (from 8.11 to 26.98%), streptomycin(SM)-resistant TB (from 50.00 to 71.43%) increased significantly (*P* < 0.05). On the contrary, the proportion of female, non-cavity, isoniazide(INH)-resistant TB (from 55.41 to 48.15%) and MR-TB (from 82.43 to 62.43%) decreased significant (*P* < 0.05). The primary drug resistance rate among female, cavity, smoking, drinking, 15 to 44 year-old TB subgroups increased by 0.16, 6.24, 20.95, 158.85, 31.49%, respectively. The percentage of primary DR-TB, RFP-resistant TB dropped significantly during 2004–2007 in Joinpoint regression model.

**Conclusion:**

The total rate of drug resistance among new TB cases showed a downward trend in Shandong, China, from 2004 to 2018. Primary drug resistance patterns were shifting from female, non-cavity, INH-resistant TB, and MR-TB groups to male, cavity, RFP/SM-resistant TB, and MDR-TB groups. Considering the rising drug resistance rate among some special population, future control of primary DR-TB in China may require an increased focus on female, cavity, smoking, drinking, or 15 to 44 year-old TB subgroups.

## Background

Drug resistance of *mycobacterium tuberculosis* (MTB), especially multidrug resistant tuberculosis (MDR-TB, defined as resistance to at least rifampin and isoniazid) has contributed to a significant health and economic burden on a global scale [1–3]. Drug-resistant tuberculosis (DR-TB) can be transmitted (primary resistance, refers to the infection with drug-resistant MTB) or develop during the course of treatment (secondary or acquired resistance) [3]. Plenty of researches [4–6] had revealed that the majority of DR-TB were primary instead of acquired drug resistance, in other words, the main mechanism of drug resistance in TB was the transmission of drug-resistant MTB strains from the existing TB patients rather than mismanagement of previous treatment episode. For instance, a molecular epidemiological study on DR-TB strains in Shanghai using highly discriminatory whole genome sequencing (WGS) found that more than 73% of MDR-TB cases were caused by primary transmission [5, 6].

According to the World Health Organization (WHO) Global TB report 2018, an estimated 10 million people developed TB in 2017, of whom 3.5% of newly diagnosed and 18% of retreated TB cases were MDR-TB, and China had the world’s second largest number of TB cases and MDR/rifampicin-resistant TB(RR-TB), comprising around 9 and 13% of the world total, respectively, behind only India (TB cases, 27%; MDR/RR-TB, 24%) [7]. Based on a national survey of drug-resistant tuberculosis (DR-TB) in China, there were 869,092 new TB cases and 54,216 previously treated TB cases in 2010, and the rate of primary and acquired MDR-TB were 5.7% (95% confidence interval [CI], 4.5–7.0) and 25.6% (95% CI, 21.5–29.8), respectively [4]. Great progress in TB control and prevention had been made in China since the implement of DOTS strategy in 1990s, and prevalence rate of TB reduced 3.4% per year [8]. However, previous studies reported that the rate of total DR-TB remained a high level, and the overall MDR rate was 6.2% in Shandong during 2007–2014, overall rifampin (RFP) resistance and rifampin monoresistance (RMR) increased at a yearly rate of 0.2 and 0.1%, respectively [9].

Understanding the burden of primary DR-TB and the factors associated with its transmission may help determine the high-risk population for drug-resistant MTB infection and develop measures for preventing transmission. Moreover, investigating the current epidemiological status of primary DR-TB in China also help to assess the effectiveness of existing TB interventions and provide guidance for TB control in future. However, few studies [4, 10] had focused on the epidemic of primary DR-TB in China. To evaluate the trend, high risk factors and epidemiological characteristics of primary DR-TB in China from 2004 to 2018, we collected 11,467 new TB cases with drug susceptibility test (DST) results for statistical analysis by applying a join-point regression model and chi-square regression for trend, and DR-TB groups were stratified by drug-resistant profiles, age, sex, smoking, drinking and cavity for further analysis.

## Materials and methods

### Ethics statement

The protocols applied in this study were approved by the Ethics Committee of Shandong Provincial Hospital, affiliated with Shandong University (SPH) and and the Ethic Committee of Shandong Provincial Chest Hospital (SPCH), China. Before analysis, patient records were anonymized and deidentified.

### Setting

This study was carried out in Shandong, a coastal province of the East China region. Shandong consisted of 17 municipalities and 140 counties (districts) with 95.8 million inhabitants at the 2010 Census. It is located at 36°24′N latitude 118°24′E longitude with an area of 157,100 km^2^. Shandong has emerged as one of the most populous and most affluent provinces in China since the late nineteenth century.

### Study population and data collection

A total of 11,467 newly diagnosed and 1981 retreated MTB cases were collected from 36 TB prevention and control institutions of Shandong Province, China, Jan 1, 2004 to Dec 31, 2018. Two province-level hospitals (SPH and SPCH), 13 municipal-level and 21 county-level local health departments were involved in the surveillance of DR-TB from 2004 to 2018. All MTB cases enrolled in our study were consecutive culture-confirmed and finished DST for first line anti-TB drugs. Demographic and clinical characteristics on age, sex, drinking, smoking, cavity, treatment history and extra-pulmonary TB were available. Smoker (or drinker) refers to those who satisfy at least one of the following two conditions: i) smoking (or drinking) for 6 months or above; ii) those who was still smoking(or drinking) or had stopped smoking (or drinking) for less than 6 months before TB diagnosis. Non-smoker and non-drinker were defined as the person who had never smoked or never drunk, respectively.

### Bacteriologic examinations and drug susceptibility testing

Each surveillance site collected two sputum samples from all eligible patients, and then sent all sputum samples to the TB Reference Laboratory of SPCH for further examination including bacteriologic culture, DST, and species identification. Isolates were inoculated into tubes of acidified Löwenstein-Jensen (LJ) medium after conventional pretreatment process and cultured at 37 °C [4]. Cultures with growing colonies were sent for further identification and DST. According to previous published protocol [11], standard traditional biochemical testings such as P-nitrobenzoic acid, 2-thiophene carboxylic acid hydrazide testing and 16S rRNA gene sequence analysis (MicroSeq ID Microbial Indentification Software(version 2.0); Applied Biosystems, Foster City, CA, USA) [12] were used to differentiate *M. tuberculosis* from other *Mycobacteria spp*.. DST of *M. tuberculosis* were performed using absolute concentration method on L-J media, and all procedures were carried out in accordance with the guideline of WHO [13]. The concentration of four first-line anti-TB drugs were as follows: 0.2 μg/mL (isoniazid, INH), 40 μg/mL(rifampin, RFP), 10 μg/mL (streptomycin, SM), 2 μg/mL (ethambutol, EMB). DST of ethionamide, fluoroquinolone, kanamycin and pyrazinamide were not routinely performed.

### Quality control

Two professionally trained investigators were independently responsible for quality assessment and data extraction; and all laboratories involved in our study regularly accepted external quality assessment of Superior TB National Reference laboratory in SPCH.

### Definitions

Drug-resistant tuberculosis (DR-TB) are classified as having acquired or primary drug resistance on the basis of a history of previous treatment [13].

Mono-resistance (MR) refers to resistance to one first-line anti-TB drug only [14].

Polydrug resistance (PDR) refers to resistance to more than one first-line anti-TB drug, other than both isoniazid and rifampicin [14].

Multidrug resistance (MDR) refers to resistance to at least both isoniazid and rifampicin [14].

### Statistical analysis

Categorical variables including age (0–14, 15–44, 45–64, 65+), sex(male or female), drinking (yes/no/unknown), smoking (yes/no/unknown), cavity (yes/no/unknown), patients type (extra-pulmonary TB/pulmonary TB) of new and relapse TB cases were calculated as counts and proportions, respectively. In addition, odds ratios (ORs) and 95% CIs for the comparisons of these characteristics were estimated between newly diagnosed susceptible and DR-TB cases, retreated susceptible and DR-TB cases, new and relapse TB cases, primary and acquired DR-TB cases. Differences in drug susceptibility profiles between primary DR M. tuberculosis and acquired drug-resistant *M. tuberculosis* isolates were analyzed using Pearson Chi-square test, and *p-*value < 0.05 was considered statistically significant. Chi-square test for trends and linear regression in line charts and stacked bar charts were used to assess the changes and temporal trend in quantity and proportions of the different resistance patterns among total DR-TB cases from 2004 to 2018.

We calculated total or annual primary drug resistance rate of tuberculosis as the number of annual primary DR-TB cases divided by the population of newly diagnosed TB cases. The overall rate and the annual rate stratified by age groups, sex, drinking (yes/no/unknown), smoking (yes/no/unknown), cavity (yes/no/unknown) and type (extra-pulmonary TB/pulmonary TB) were estimated as well. Indicators including annual percent changes (APCs) and average annual percent changes (AAPCs) were calculated from 2004 to 2018 inclusive on the basis of a joinpoint regression model. Each segment described a short-term trend (APC). Long-term trends over the entire study period are AAPCs and were estimated as the weighted average of the short-term APCs, with the weights equal to the length of the short-term line segment [15]. Two-sided *t*-tests at *p* < 0.05 were used to test whether AAPCs and APCs were statistically significantly different from zero; 95% *CI* for each segment were calculated. If the AAPC was within one segment, the t-distribution was used. Otherwise, the normal (z) distribution was used [15–17]. A non-significant (*p* ≥ 0.05) APC was described as stable while a significant (*p* < 0.05) positive or negative APC was termed as increase or decrease. All analyses were carried out using SPSS software (version 20.0) and the Joinpoint Regression Software (version 4.3.1.0).

## Results

### Patients’ characteristics

Table 1 illustrates the baseline characteristics of the study participants. A total of 11,467 new and 1981 relapse TB cases were reported during the period from Jan 1, 2004 to Dec 31, 2018 in Shandong, China, of which 2173 (18.95%) and 505 (23.24%) were drug-resistant TB cases, respectively, *P* < 0.01. Among these primary DR-TB cases, there were more males (83.06% vs 16.94%), non-smokers (40.73% vs 13.90%), non-drinkers (42.84% vs 11.50%), pulmonary TB (99.68% vs 0.32%). In addition, most primary DR-TB cases (2168, 99.77%) were from three age groups: 15–44 years (930, 42.80%), 45–64 years (728, 33.50%), > 65 years (486, 22.37%). The distribution of age, sex, smoking, et al. among acquired DR-TB cases were similar to primary DR-TB cases (Table 1).

**Table 1:** Demographic and clinical characteristics of new and relapse TB patients, Shandong, China, 2004–2018^a^

Characteristics	New case,no.,(%) *n* = 11,467					Relapse case,no.,(%) *n* = 1981					New case vs Relapse case (control)		Primary DR-TB vs Acquired DR-TB	
	Total *n* = 11,467	DR-TB *n* = 2173	Susceptible TB *n* = 9294	OR (95% CI)	*P* value	Total *n* = 1981	DR-TB *n* = 505	Susceptible TB *n* = 1476	OR (95% CI)	*P* value	OR (95% CI)	*P* value	OR (95% CI)	*P* value
Age (years)														
0–14	26(0.23)	5(0.23)	21(0.23)	–	–	4(0.20)	1(0.20)	3(0.20)	–	–	–	–	–	–
15–44	4796(41.82)	930(42.80)	3866(41.60)	Ref.	Ref.	719(36.29)	191(37.82)	528(35.77)	Ref.	Ref.	Ref.	Ref.	Ref.	Ref.
45–64	3655(31.87)	728(33.50)	2927(31.49)	1.034(0.928–1.152)	0.546	652(32.91)	182(36.04)	470(31.84)	1.070(0.844–1.358)	0.575	0.840(0.750–0.942)	0.003	0.822(0.656–1.029)	0.087
> 65	2890(25.20)	486(22.37)	2404(25.87)	0.840(0.745–0.948)	0.005	509(25.69)	101(20.00)	408(27.64)	0.684(0.521–0.900)	0.007	0.851(0.753–0.962)	0.010	0.988(0.758–1.288)	0.930
Sex														
Female	2153(18.78)	368(16.94)	1785(19.21)	Ref.	Ref.	357(18.02)	82(16.24)	275(18.63)	Ref.	Ref.	Ref.	Ref.	Ref.	Ref.
Male	9311(81.20)	1805(83.06)	7506(80.76)	1.166(1.031–1.32)	0.014	1556(78.55)	402(79.60)	1154(78.18)	1.168(0.891–1.533)	0.262	0.992(0.876–1.123)	0.902	1.001(0.769–1.301)	0.997
Cavity														
No	6214(54.19)	1142(52.55)	5072(54.57)	Ref.	Ref.	1156(58.35)	251(49.70)	905(61.31)	Ref.	Ref.	Ref.	Ref.	Ref.	Ref.
Yes	3854(33.61)	758(34.88)	3096(33.31)	1.087(0.982–1.204)	0.108	637(32.16)	191(37.82)	446(30.22)	1.544(1.240–1.924)	*P* < 0.001	1.126(1.024–1.250)	0.027	0.872(0.707–1.075)	0.201
Unknown	1399(12.20)	273(12.56)	1126(12.12)	1.077(0.930–1.247)	0.324	188(9.49)	63(12.48)	125(8.47)	1.817(1.302–2.537)	*P* < 0.001	1.384(1.174–1.632)	*P* < 0.001	0.952(0.701–1.294)	0.755
Smoking														
No	4809(41.94)	885(40.73)	3924(42.22)	Ref.	Ref.	1507(76.07)	358(70.89)	1149(77.85)	Ref.	Ref.	Ref.	Ref.	Ref.	Ref.
Yes	1660(14.48)	302(13.90)	1358(14.61)	0.986(0.853–1.139)	0.849	371(18.73)	105(20.79)	266(18.02)	1.267(0.982–1.635)	0.069	1.402(1.235–1.591)	*P* < 0.001	1.163(0.903–1.5)	0.242
Unknown	4998(43.59)	986(45.38)	4012(43.17)	1.057(0.955–1.169)	0.288	103(5.20)	42(8.32)	61(4.13)	2.210(1.466–3.331)	*P* < 0.001	15.206(12.406–18.638)	*P* < 0.001	9.497(6.812–13.24)	*P* < 0.001
Drinking														
No	5032(43.88)	931(42.84)	4101(44.13)	Ref.	Ref.	1552(78.34)	381(75.45)	1171(79.34)	Ref.	Ref.	Ref.	Ref.	Ref.	Ref.
Yes	1398(12.19)	250(11.5)	1148(12.35)	0.959(0.822–1.119)	0.597	315(15.90)	79(15.64)	236(15.99)	1.029(0.778–1.361)	0.842	1.369(1.196–1.566)	*P* < 0.001	1.295(0.979–1.713)	0.070
Unknown	5037(43.93)	992(45.65)	4045(43.52)	1.080(0.978–1.193)	0.128	114(5.75)	45(8.91)	69(4.67)	2.004(1.353–2.969)	0.001	13.628(11.233–16.548)	*P* < 0.001	9.021(6.540–12.444)	*P* < 0.001
Type														
Extrapulmonary TB	40(0.35)	7(0.32)	33(0.36)	Ref.	Ref.	17(0.86)	7(1.39)	10(0.68)	Ref.	Ref.	Ref.	Ref.	Ref.	Ref.
Pulmonary TB	11,427(99.65)	2166(99.68)	9261(99.64)	1.103(0.484–2.496)	0.815	1964(99.14)	498(98.61)	1466(99.32)	0.485(0.184–1.282)	0.145	2.473(1.399–4.370)	0.002	4.349(1.519–12.456)	0.006

^a^*OR* Odds ratio, *TB* Tuberculosis, *DR-TB* Drug-resistant tuberculosis

Compared with newly diagnosed susceptible TB cases, primary DR-TB cases were more likely to be female (OR: 1.166, 95%CI:1.031–1.320) and less likely to be aged more than 65(OR: 0.840, 95%CI:0.745–0.948). Among retreated TB cases, factors associated with acquired resistance were baseline cavitary diseases (OR:1.544, 95%CI:1.24–1.924) and > 65 years (OR:0.684, 95%CI:0.521–0.900). New TB were less likely to be aged 45–64 years old (OR:0.84, 95%CI: 0.750–0.942) or > 65 years old (OR:0.851, 95%CI: 0.753–0.962) when compared with relapse TB cases. Moreover, patients with pulmonary TB had a statistically significant higher risk of primary drug resistance (vs acquired drug resistance) than those with extra-pulmonary TB (OR:4.349, 95%CI:1.519–12.456) (Table 1).

### Resistance patterns

Among 11,467 newly diagnosed and 1981 retreated TB clinical isolates, the number and proportion of any resistance to first-line drugs including INH, RFP, EMB, SM were 1232(10.74%) and 333(16.81%), 508(4.43%) and 223(11.26%), 174(1.52%) and 68(3.43%), 1482(12.92%) and 329(16.61%), respectively; new TB cases had a lower rate of DR-TB(18.95% vs 23.24%, *P* < 0.01), MDR-TB(3.19% vs 8.43%, *P* < 0.01), PDR-TB(4.12% vs 5.55%, *P* < 0.01) than relapse TB cases, but almost the same in MR-TB (11.56% vs 11.36%, *P* > 0.05). Of 1326 primary MR-TB cases, SM was associated with the highest rate of resistance (6.41%,758), followed by INH (3.88%, 445), RFP (0.78%, 90), and EMB (0.17%, 20). There were four main types of primary MDR-TB, for instance, MDR1 (INH + RFP), MDR2 (INH + RFP + EMB), MDR3 (INH + RFP + EMB + SM), and MDR4 (INH + RFP + SM), which accounted for 0.71%(81), 0.13%(15), 0.72%(83), 1.45%(166) respectively. Similarly, primary PDR-TB mainly consisted of PDR1 (INH + EMB, 0.12%, 14), PDR2 (INH + SM, 3.41%, 391), PDR3 (RFP + EMB, 0.06, 7), PDR4 (RFP + SM, 0.33%, 38), PDR5 (INH + EMB + SM, 0.14%, 16) (Table 2).

**Table 2:** Primary and acquired drug resistance profiles of *Mycobacterium tuberculosis*, Shandong, China, 2004–2018^a^

Drug resistance	New case, no., (%) *n* = 11,467	Relapse case, no., (%) *n* = 1981	*P* value
DR-TB	2173 (18.95)	505 (23.24)	*P* < 0.001
Any resistance to first-line drugs			
INH	1232(10.74)	333(16.81)	*P* < 0.001
RIF	508(4.43)	223(11.26)	*P* < 0.001
EMB	174(1.52)	68(3.43)	*P* < 0.001
SM	1482(12.92)	329(16.61)	*P* < 0.001
MR-TB (Total)	1326(11.56)	225(11.36)	0.791
INH	445(3.88)	82(4.14)	0.584
RIF	90(0.78)	29(1.46)	0.003
EMB	20(0.17)	9(0.45)	0.013
SM	758(6.61)	99(5.00)	0.007
Others	13(0.11)	6(0.30)	0.038
MDR-TB (Total)	366(3.19)	167(8.43)	*P* < 0.001
MDR1:INH + RFP	81(0.71)	37(1.87)	*P* < 0.001
MDR2:INH + RFP + EMB	15(0.13)	6(0.30)	0.073
MDR3:INH + RFP + EMB + SM	83(0.72)	33(1.67)	*P* < 0.001
MDR4:INH + RFP + SM	166(1.45)	76(3.84)	*P* < 0.001
Others	21(0.18)	15(0.76)	*P* < 0.001
PDR-TB	473(4.12)	110(5.55)	0.004
PDR1:INH + EMB	14(0.12)	1(0.05)	0.713
PDR2:INH + SM	391(3.41)	77(3.89)	0.285
PDR3:RFP + EMB	7(0.06)	3(0.15)	0.173
PDR4:RFP + SM	38(0.33)	19(0.96)	*P* < 0.001
PDR5:INH + EMB + SM	16(0.14)	5(0.25)	0.240
Others	7(0.06)	5(0.25)	0.008

^a^*EMB* ethambutol, *INH* Isoniazid, *RFP* Rifampin, *SM* Streptomycin, *TB* Tuberculosis, *DR-TB* Drug-resistant tuberculosis, *MR-TB* Mono-resistant tuberculosis, *MDR-TB* Multi-resistant tuberculosis, *PDR-TB* Polydrug resistant tuberculosis

### Disparity in the proportion of primary DR-TB subgroups

**Fig. 1:**
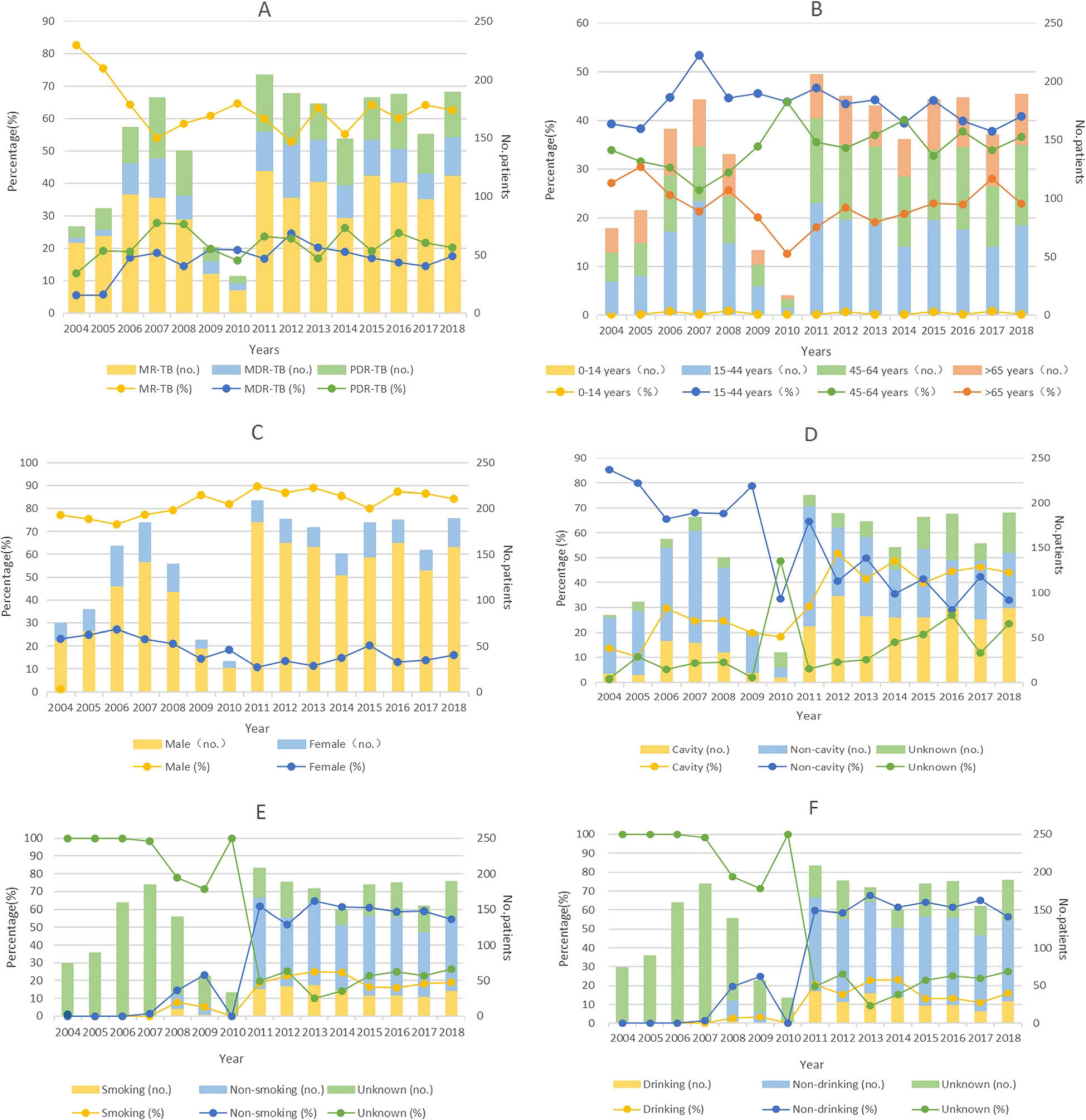
Trends for the quantity and proportions of different subgroups among total primary DR-TB cases, Shandong, China, 2004–2018*. **a** Trends for MR-TB, MDR-TB, PDR-TB among total primary cases of TB; **b** Trends for primary DR-TB cases of different age (0–14, 15–44, 45–64, 65+); **c** Trends for primary DR-TB cases of different sex(male or female); **d** Trends for primary DR-TB cases with or without cavity; **e** Trends for primary DR-TB cases with or without smoking history; **f** Trends for primary DR-TB cases with or without drinking history; The proportions of each subgroups were calculated as follows: (the quantity of each subgroups/ the quantity of total primary DR-TB subgroups in the same year)*100%. The χ^2^ and linear regression results are shown in Table 5 in Appendix. TB, tuberculosis; DR-TB, drug-resistant tuberculosis; MR-TB, mono-resistant tuberculosis; MDR-TB, multi-resistant tuberculosis; PDR-TB, polydrug resistant tuberculosis. EMB, ethambutol; INH, isoniazid; RFP, rifampin; SM, streptomycin

**Fig. 2:**
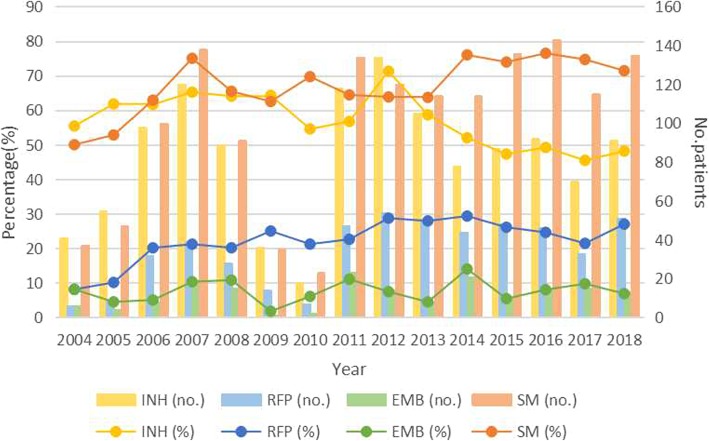
Overall first-line drug resistance for INH, RIF, EMB, and SM in primary cases of tuberculosis in Shandong China, 2004–2018. The proportions of INH-, RIF-, EMB-, and SM-resistance were calculated as follows: (the quantity of each subgroups/ the quantity of total primary DR-TB subgroups in the same year)*100%. The χ^2^ and linear regression results are shown in Table 5 in Appendix. EMB, ethambutol; INH, isoniazid; RFP, rifampin; SM, streptomycin

As shown in Figs. 1a-f and 2, primary DR-TB cases was sub-divided into different subgroups according to the types of drug resistance (MR-TB/MDR-TB/PDR-TB, INH/RFP/SM/EMB-resistance), age (0–14, 15–44, 45–64, 65+), sex (male or female), drinking history (yes/no/unknown), smoking history (yes/no/unknown), cavity (yes/no/unknown), and the amount and proportions of each subgroup among the total primary DR-TB cases varied every year since 2004 to 2018. Among all primary DR-TB cases, the percentage of MDR-TB ([*R*^*2*^ = 0.2034] from 5.41 to 17.46%; *χ2* test for trends: *χ2* = 5.376, *P* = 0.020), males ([*R*^*2*^ = 0.4834] from 77.03 to 84.13%; *χ2* test for trends: *χ2* = 12.570, *P < 0.001*), cavity ([*R*^*2*^ = 0.7022] from 13.51 to 43.92%; *χ2* test for trends: *χ2* = 120.53, *P < 0.001*), RFP-resistance ([*R*^*2*^ = 0.5355] from 8.11 to 26.98%; *χ2* test for trends: *χ2* = 16.785, *P < 0.001*), SM-resistance ([*R*^*2*^ = 0.5365] from 50.00 to 71.43%; *χ2* test for trends: *χ2* = 22.076, *P < 0.001*) increased significantly from 2004 to 2018. On the contrary, the proportion of females, non-cavity, INH ([*R*^*2*^ = 0.4269] from 55.41 to 48.15%; *χ2* test for trends: *χ2* = 14.725, *P < 0.001*) and MR-TB ([*R*^*2*^ = 0.178] from 82.43 to 62.43%; *χ2* test for trends: *χ2* = 6.287, *P* = 0.012) decreased significantly (*P* < 0.05) (Table 5 in Appendix).

### Total and annul primary drug resistance rate

Table 3 illustrates the overall and annul primary drug resistance rate of various TB subgroups from 2004 to 2018. During the study period, the primary drug resistance rate among female, cavity, smoking, drinking, 15 to 44 year-old TB subgroups increased by 0.16, 6.24, 20.95, 158.85, 31.49%, and varied from 20.24 to 20.27%, from 20.83 to 22.13%, from 21.57 to 26.09%, from 10.26 to 26.55%, 18.59 to 24.44%, respectively; meanwhile the rate of DR-TB among male, non-cavity, non-smoking, non-drinking, 45 to 64 year-old, > 65 year-old TB subgroups reduced by 16.62, 26.34, 15.79, 22.18, 35.01, 36.66% respectively, decreasing from 20.24 to 20.27%, 24.90 to 18.34%, 21.57 to 26.09%, 25.00 to 19.45%, 32.47 to 21.10%, and 28.17 to 17.84%, respectively. In addition, the rate of MDR-TB, PDR-TB, RFP-resistant TB, and SM-resistant TB among new TB cases increased from 1.32 to 3.73%, 2.96 to 4.30%, 1.97 to 5.77%, and 12.17 to 15.27%, with a percentage change of 183.71, 45.20, 192.31, 25.47%, respectively; the rates of MR-TB, INH-resistant TB, EMB-resistant TB, and total DR-TB dropped by 33.48, 23.67, 25.49, 12.17%, respectively, with a decrease from 20.07 to 13.35%, 13.49 to 10.29%, 1.97 to 1.47%, respectively.

**Table 3:** The temporal change trend of primary drug resistance rate among newly diagnosed TB cases in Shandong, China, 2004–2018

Characteristics	Primary drug resistance rate(%)															Change^a^ (%)
	2004	2005	2006	2007	2008	2009	2010	2011	2012	2013	2014	2015	2016	2017	2018	2005–2017
DR-TB (Total)	24.34	17.91	19.73	17.81	19.72	13.97	30.28	19.75	17.99	17.83	17.26	18.66	19.10	19.44	21.38	−12.17
Type																
MR-TB	20.07	13.48	12.66	9.58	11.49	8.48	18.35	11.59	9.47	11.25	9.44	11.97	11.44	12.37	13.35	−33.48
MDR-TB	1.32	1.01	3.35	3.29	2.84	2.74	5.50	3.23	4.40	3.59	3.22	3.14	2.96	2.78	3.73	183.71
PDR-TB	2.96	3.42	3.72	4.94	5.39	2.74	4.59	4.56	4.11	2.99	4.49	3.55	4.70	4.17	4.30	45.20
Age (years)																
15–44	18.59	15.53	19.35	21.03	19.93	14.12	29.17	19.55	17.88	18.94	16.08	21.49	19.58	20.49	24.44	31.49
45–64	32.47	19.31	19.92	16.10	18.69	14.39	41.18	21.99	19.34	19.64	19.93	18.35	21.67	20.00	21.10	−35.01
> 65	28.17	20.30	19.80	14.72	20.23	12.22	10.53	17.05	16.40	13.77	15.82	15.11	15.50	17.77	17.84	−36.66
Sex																
Male	25.91	17.49	19.21	18.37	21.11	15.95	27.84	20.95	17.91	18.47	17.95	18.08	19.93	19.76	21.60	−16.62
Female	20.24	19.30	21.29	16.15	15.85	8.16	50.00	13.33	18.52	13.99	14.10	21.39	14.91	17.65	20.27	0.16
Cavity																
Yes	20.83	12.00	25.27	18.29	20.73	10.38	35.29	18.53	25.39	18.59	18.34	19.73	20.70	20.40	22.13	6.24
No	24.90	19.51	17.72	17.46	19.26	15.71	25.00	20.55	13.10	17.08	15.63	19.24	15.43	21.17	18.34	−26.34
Smoking																
Yes					21.57	13.04		15.00	15.09	19.23	18.78	19.48	16.67	21.05	26.09	20.95
No				23.08	15.63	16.67		21.23	16.67	17.24	16.11	19.18	19.37	19.12	19.43	−15.79
Drinking																
Yes					10.26	11.76		18.86	12.39	19.34	21.34	19.35	16.45	15.74	26.55	158.85
No				25.00	19.29	16.67		19.53	17.52	17.41	15.59	19.44	19.39	20.16	19.45	−22.18
First-line drugs																
INH	13.49	11.07	12.16	11.62	12.62	8.98	16.51	11.21	12.82	10.46	8.98	8.82	9.40	8.84	10.29	−23.67
RFP	1.97	1.81	3.97	3.78	3.97	3.49	6.42	4.46	5.17	4.98	5.06	4.87	4.70	4.17	5.77	192.31
EMB	1.97	0.80	0.99	1.84	2.13	0.25	1.83	2.18	1.34	0.80	2.42	1.01	1.53	1.89	1.47	−25.49
SM	12.17	9.46	12.41	13.36	12.91	8.73	21.10	12.73	11.48	11.35	13.12	13.79	14.61	14.52	15.27	25.47

Primary drug resistance rate(%) were calculated as follows: (the quantity of each DR-TB subgroups/ the quantity of corresponding primary TB cases (total) in the same year)*100%, for example, primary drug resistance rate(%) of female TB cases in 2018 = the quantity of female DR-TB cases in 2018/ the quantity of total female primary TB cases in 2018) * 100%

Joinpoint regression of these crude ratios were shown in Table 4

*TB* Tuberculosis, *DR-TB* Drug-resistant tuberculosis, *MR-TB* Mono-resistant tuberculosis, *MDR-TB* Multi-resistant tuberculosis, *PDR-TB* Polydrug resistant tuberculosis, *EMB* Ethambutol, *INH* Isoniazid, *RFP* Rifampin, *SM* Streptomycin

^a^The % changes were calculated as follows: (incidence in 2018-incidence in 2005)/incidence in 2005

### Temporal trends and Joinpoint regression model

The Joinpoint regression analysis (Table 4) revealed that total primary MR-TB rate had decreased during 2004–2007 (APC = -20.6% [95%CI: − 33.7, − 4.9], Z = -3.00, *P* < 0.01). In contrast, total primary MDR-TB rate had increased during 2004–2006 (APC = 57.9% [95%CI: 0.3, 148.7], *Z* = 2.40, *P* < 0.01). The rate of INH resistant TB showed a significant decline in the trend with an APC of − 2.3% during 2004–2018(*P* < 0.01), while the rate of RFP resistant TB were rising significantly during 2004–2010 (APC = 16.90% [95%CI: 5.8, 29.1], *Z* = 3.50, *P* < 0.01).

**Table 4:** Annual percentage change in primary drug-resistant rate of *Mycobacterium tuberculosis* in Shandong, China, 2004–2018

Variables	Phases	APC	Test Statistic (t)	Prob > |t|*
Type				
DR-TB (Total)	2004–2007	−9.2(− 21.2,4.6)	−1.60	0.20
	2007–2010	6.2(−20.1,41.1)	0.50	0.60
	2010–2018	−0.9(−4.0,2.2)	− 0.70	0.50
MR-TB	2004–2007	− 20.6*(− 33.7,-4.9)	−3.00	0.00
	2007–2010	7.5(−25.1,54.3)	0.50	0.60
	2010–2018	−0.2(−4,3.8)	− 0.10	0.90
MDR-TB	2004–2006	57.9*(0.3148.7)	2.40	*P* < 0.001
	2006–2010	11.4(−11.3,39.7)	1.10	0.30
	2010–2018	−3.4(− 8,1.6)	−1.60	0.10
PDR-TB	2004–2007	16.7(−17.6,65.4)	1.00	0.30
	2007–2013	−3.8(− 17.7,12.4)	−0.60	0.60
	2013–2018	4.1(− 10.9,21.6)	0.60	0.60
Age (years)				
15–44	2004–2010	3.8(−4.1,12.4)	1.10	0.30
	2010–2014	−5.3(−25.2,19.9)	−0.50	0.60
	2014–2018	8.4(− 6.6,25.8)	1.30	0.20
45–64	2004–2007	−20.5*(−33.2,-5.3)	− 3.10	*P* < 0.001
	2007–2010	18.1(− 16.8,67.7)	1.10	0.30
	2010–2018	−3.2(−6.8,0.6)	−2.00	0.10
> 65	2004–2009	−12.7*(−21.8,-2.4)	−2.70	0.00
	2009–2018	3.3(−1.3,8.1)	1.60	0.10
Sex				
Female	2004–2008	−6.4(−39.8,45.4)	−0.30	0.70
	2008–2018	1.2(−9.2,12.8)	0.20	0.80
Male	2004–2006	−10.3(−43.5,42.5)	−0.50	0.60
	2006–2018	0.4(−2.3,3.2)	0.40	0.70
Cavity				
No	2004–2006	−11.9(−55.5,74.7)	− 0.40	0.70
	2006–2014	−1.3(−9.9,8.2)	−0.30	0.70
	2014–2018	3.5(−16.6,28.5)	0.40	0.70
Yes	2004–2010	3.2(−9.6,17.9)	0.50	0.60
	2010–2018	−0.1(−8.3,8.9)	0.00	1.00
Smoking				
No	2008–2018	1.6(−0.5,3.8)	1.80	0.10
Yes	2008–2011	−7.2(−41,45.9)	−0.40	0.70
	2011–2018	7.1*(0.8,13.8)	2.90	*P* < 0.001
Drinking				
No	2008–2014	−1.2(−4.9,2.6)	−0.80	0.40
	2014–2018	4.5(−2.7,12.1)	1.60	0.20
Yes	2008–2018	6.7*(1.6,12.1)	3.00	*P* < 0.001
First-line drugs				
INH	2004–2018	−2.3*(−4.3,-0.3)	− 2.50	*P* < 0.001
RFP	2004–2010	16.9*(5.8,29.1)	3.50	*P* < 0.001
	2010–2018	−1.1(−7.3,5.4)	−0.40	0.70
EMB	2004–2009	−6.9(−30.5,24.6)	−0.50	0.60
	2009–2018	6(−5.9,19.4)	1.10	0.30
SM	2004–2018	1.8(−0.7,4.4)	1.50	0.10

*APC* Annual percent change, *TB* Tuberculosis, *DR-TB* Drug-resistant tuberculosis, *MR-TB* Mono-resistant tuberculosis, *MDR-TB* Multi-resistant tuberculosis, *PDR-TB* Polydrug resistant tuberculosis, *EMB* Ethambutol, *INH* Isoniazid, *RFP* Rifampin, *SM* Streptomycin

*P < 0.05

The primary DR-TB rate among smoking and drinking subgroups had been on the rise during 2011–2018 (APC = 7.1% [95%CI: 0.8, 13.8], *Z* = 2.90, *P* < 0.01) and 2008–2018 (APC = 6.7% [95%CI: 1.6,12.1], Z = 3.00, *P* < 0.01), respectively. Of 45–64 year-old TB group, the primary DR-TB rate decline during 2004–2007(APC = -20.5% [95%CI: − 33.2,-5.3], *Z* = -3.10, *P* < 0.01). DR-TB among elderly TB cases (> 65 years) showed a sharply drop during 2004–2009 (APC = -12.7% [95%CI: − 21.8,-2.4], *Z* = -2.70, *P* < 0.01).

## Discussion

Our study had enrolled 11,467 newly diagnosed TB cases with DST results at 36 TB surveillance centers across Shandong, China from 2004 to 2018 to evaluate the epidemiology and high risk factors of primary TB drug resistance, and we found that ongoing transmission of drug resistant MTB remained to be the major mechanism of DR-TB in China, approximately one fifth of new TB cases were primary DR-TB while 1.3–4.4% were MDR-TB, slightly differed from previous researches in Zhejiang, Jiangsu, Heilongjiang, China [10, 18–20]. Trends in annual prevalence of primary DR revealed that the rates of MDR-TB (from 1.32 to 3.73%), PDR-TB (from 2.96 to 4.30%) began to increase slowly along with that of MR-TB (from 20.07 to 13.35%) decreasing, However, MR-TB still dominated the drug resistance rate. In a similar way the proportion of these three sub-types of DR-TB changed during our study. The increment in both MDR-TB and PDR-TB alarmed us that it would continue to be a tough challenge to TB elimination strategy in China [4, 21].

According the temporal trend in proportions of different primary DR-TB subgroups each year, we pointed out that primary resistance patterns were shifting from female, non-cavity, INH resistant TB, and MR-TB groups to male, cavity, RFP/SM resistant TB, and MDR-TB groups in Shandong China from 2004 to 2018, and more than two thirds were male or had a resistance to SM, but more than half of the present primary DR-TB cases were still MR-TB. The percentage of males among MDR-TB cases were more than 70%, which was consistent with previous researches in Bangladesh [22], Mozambique [23], et al., but in contrast with Ethiopia [24] and Lianyungang city, China [25] where females were in majority. The major reasons for this phenomenon were that with genuine gender or behavioral differences [25], men had a significant higher risk of contracting and dying from TB than women [26], and it was reported by WHO that TB incidence around China in 2017 for male patients (67.5%) was twice as much as females (32.5%) [7]. In accordance with Li D [27] and Yeom’s [28] results, we found that cavities had turned to be more common for primary DR TB since 2012 in China. Low effectiveness of anti-TB drug sensitivity towards primary DR-TB, limited drug penetration into cavities, specific microorganism virulence and immune status of patients may contribute to this phenomenon [27–29]. However, another reason the more important may be the imbalance between compensatory evolution and compensation cost of drug resistant MTB strains [30, 31]. Four out of ten primary DR-TB cases were in the age group of 15–44 years, followed by groups of 45–64 years (33.5%) and > 65 years (22.37%), suggesting that DR-TB prevention and control should focus more on young and middle aged patients. In recent years, TB patients who were alcohol users and smokers were associated with a higher annual primary DR-TB prevalence than those who were non-smokers and non-drinkers. However, when total TB cases across 2004–2018 were involved in bivariate analysis, personal behaviours like smoking and alcohol use were no longer predictors of primary DR-TB on bivariate analysis, in contrast to findings in India [32] and Botswana [33] where alcohol use was a risk factor for MDR-TB, but consistent with studies in Nepal [34], indicating that it may be distinguished by regions and phases.

Directly observed treatment, short-course (DOTS) strategy had been put forward in China since 1990s, and was expanded to cover all smear-positive and smear-negative TB patients since 2005 instead of previous policy that only patients diagnosed with smear-positive or severe smear-negative TB were available to free treatment [35, 36]. As a consequence, TB prevalence has declined sharply from 1990 to 2010 [36, 37], in accordance with which both the percentage of primary DR-TB, RFP resistant TB and the drug resistant rate of 45–64 year-old, > 65 year-old or female group dropped significantly during 2004–2007. Possible reason for this trend was the reduction of acquired DR-TB due to the free treatment policy in China (*35–37)*, in other words, the source of primary DR-TB had been controlled effectively. An obvious increment in primary MDR-TB and RFP resistant TB during 2004–2008 had been observed, and one hypothesis was that MDR/RR-TB were more difficult to treat than drug-susceptible TB and other types of DR-TB, thus it was hard to prevent the newly transmission of previous MDR/RR-TB as well [4, 7, 38]. Furthermore, evidence has shown that substantial costs associated with TB diagnosis and treatment remained a heavy financial burden for TB patients in spite of the “free” TB care policy [35]. Other mechanisms remained to be further explored. Another issue of concern was that people with smoking or drinking habits tended to be more susceptible to infection of drug-resistant MTB [39, 40], consistent to our research since 2008. In comparison with never-smokers, current smokers had an excess risk of pulmonary TB (adjusted HR, 2.87; 95% CI, 2.00–4.11; *p* < 0.001) [40].

Our study had several advantages. First, our study covered all DST data of Shandong province from 2004 to 2018, which is the second largest province in China with a population of nearly 90 million, thus the findings of our research were more likely to be rolled out nationally. Second, we distinguished primary DR-TB from acquired DR-TB, and the total TB or DR-TB population were stratified by various factors including sex, age, smoking history, drinking history, cavity and so on, other than most previous epidemiological researches in DR-TB that considered new and relapse DR-TB together [1, 2, 9, 10]. Third, we proposed a Joinpoint regression model to describe the temporal trend and turning point of TB control in Shandong, China.

There were also some limitations of our study. First, drug sensitive tests were not routinely carried out among TB cases, thus our data from TB monitoring stations were affected by screening intensity and local medical conditions, and could be overestimated due to selection bias. Second, diversities in technical levels and experimental conditions in different TB monitoring stations may contributed to unavoidable bias. Third, DST for second-line anti-TB drugs were seldom performed by TB monitoring stations in Shandong, China, thus the epidemiology of primary resistance to second-line anti-TB drugs and extensive drug-resistant TB (XDR-TB) could not be estimated.

## Conclusion

Our study had described the epidemiological features and temporal trend of primary DR-TB in Shandong province, China from 2004 to 2018 to evaluate the current situation of TB prevention and control. We found that the proportion of primary DR-TB and MR-TB had reduced by more than 12% since 2004, and were 21.38, 13.35% in 2018 respectively. Moreover, primary drug resistance patterns were shifting from female, non-cavity, INH resistant TB, and MR-TB groups to male, cavity, RFP/SM resistant TB, and MDR-TB groups, even more than two thirds were male or had a resistance to SM, but more than half of the present primary DR-TB cases were still MR-TB. This study indicates the DOTS strategy and “free” policy in China had achieved some effects in TB control, but considering the increment of drug resistance rate among some special population, more attention should be focused on female, cavity, smoking, drinking, 15 to 44 year-old TB subgroups. In addition, more project support, high-quality training for medical staff and enhanced public awareness of TB prevention and control are also necessary for TB elimination goal in China.

## Data Availability

Data can be available through contact with the corresponding author.

## Appendix

**Table 5:** Changes in proportions of different primary drug-resistant *mycobacterium tuberculosis* subgroups, Shandong, China, 2004–2018^a^

Subgroups	*X* ^*2*^	*P* value	*R* ^*2*^	X-coefficient	SE
MR-TB (%)	6.287	0.012	0.1780	− 0.0073	0.6852
MDR-TB (%)	5.376	0.020	0.2034	0.0051	0.1221
PDR-TB (%)	0.933	0.334	0.0505	0.0022	0.1927
15–44 years(%)	0.673	0.412	0.0822	−0.0026	0.4504
45–64 years(%)	2.837	0.092	0.2046	0.0045	0.3077
> 65 years(%)	0.506	0.477	0.0399	−0.0020	0.2402
Male (%)	12.570	*P* < 0.001	0.4834	0.0081	0.7595
Female (%)	12.570	*P* < 0.001	0.4834	−0.0081	0.2405
Cavity (%)	120.530	*P* < 0.001	0.7022	0.0255	0.1202
Non-cavity (%)	120.530	*P* < 0.001	0.7244	−0.3673	0.8358
Smoking (%)	0.392	0.531	0.6400	0.0179	−0.0268
Non-smoking (%)	0.392	0.531	0.7506	0.0551	−0.0996
Drinking (%)	0.194	0.659	0.5352	0.0147	−0.0229
Non-drinking (%)	0.194	0.659	0.7634	0.0571	−0.1006
INH (%)	14.725	*P* < 0.001	0.4269	−0.0112	0.6600
RFP (%)	16.785	*P* < 0.001	0.5355	0.0101	0.1416
EMB (%)	0.746	0.388	0.0300	0.0012	0.0659
SM (%)	22.076	*P* < 0.001	0.5365	0.0133	0.5620

INH (%), RFP (%), EMB (%), and SM (%) refer to the proportion of primary INH/RFP/EMB/SM resistant TB among total primary DR TB cases, respectively

^a^*EMB* Ethambutol, *INH* Isoniazid, *RIF* Rifampin, *SM* Streptomycin, *TB* Tuberculosis

*MR-TB* Mono-resistant tuberculosis, *MDR-TB* Multi-resistant tuberculosis, *PDR-TB* Polydrug resistant tuberculosis, *EMB* Ethambutol, *INH* Isoniazid, *RFP* Rifampin, *SM* Streptomycin

## Abbreviations

AAPCs: Average annual percent changes
APCs: Annual percent changes
CI: Confidence interval
DR-TB: Drug-resistant tuberculosis
DST: Drug susceptibility test
EMB: Ethambutol
INH: Isoniazid
MDR-TB: Multi-resistant tuberculosis
MR-TB: Mono-resistant tuberculosis
MTB: *Mycobacterium tuberculosis*
ORs: Odds ratios
PDR-TB: Polydrug resistant tuberculosis
RFP: Rifampin
RR-TB: Rifampin-resistant tuberculosis
SM: Treptomycin
TB: Tuberculosis
WGS: Whole genome sequencing
WHO: World health organization
XDR-TB: Extensive drug resistant TB
